# Outbreak of *Leishmania braziliensis* Cutaneous Leishmaniasis, Saül, French Guiana

**DOI:** 10.3201/eid2105.141181

**Published:** 2015-05

**Authors:** Guillaume Martin-Blondel, Xavier Iriart, Fouad El Baidouri, Stéphane Simon, Deborah Mills, Magalie Demar, Thierry Pistone, Thomas Le Taillandier, Denis Malvy, Jean-Pierre Gangneux, Pierre Couppie, Wendy Munckhof, Bruno Marchou, Christophe Ravel, Antoine Berry

**Affiliations:** Toulouse University Hospital, Toulouse, France (G. Martin-Blondel, X. Iriart, T. Le Taillandier, B. Marchou, A. Berry);; INSERM UMR1043, Toulouse, France (G. Martin-Blondel, X. Iriart, A. Berry);; French Reference Centre on Leishmaniasis, Montpellier, France (F. El Baidouri, C. Ravel);; University of the French West Indies and Guiana, Cayenne, France (S. Simon);; Travel Medicine Alliance, Brisbane, Queensland, Australia (D. Mills);; Cayenne Hospital, Cayenne (M. Demar, P. Couppie);; Bordeaux University Hospital, Bordeaux, France (T. Pistone, D. Malvy);; Rennes University Hospital, Rennes, France (J.-P. Gangneux);; University of Queensland, Brisbane (W. Munckhof)

**Keywords:** leishmaniasis, cutaneous, Leishmania braziliensis, French Guiana, disease outbreaks, Amazonian rainforest, zoonoses, vector-borne infections, parasites

**To the Editor:** New World cutaneous leishmaniasis (CL), a zoonotic disease, is increasingly seen among travelers returning from Latin American countries, particularly from Bolivia, Belize, and French Guiana (*1*). The epidemiology of CL in the Americas is heterogeneous and has complex variations in transmission cycles, reservoir hosts, and sandfly vectors. Changing human activities that affect these factors may have resulted in the emergence of species with distinct pathogenic potentials and responses to therapy. In the Guianan ecoregion complex, leishmaniasis is endemic, and 5 coexisting *Leishmania* parasite species are known to infect humans: *L. guyanensis, L. braziliensis, L. amazonensis, L. naiffi*, and *L. lainsoni*. Among these species, *L. guyanensis* accounts for ≈85% of CL cases (*2*).

We report an outbreak of 7 cases of *L. braziliensis* CL that occurred among 24 scientists who participated in a field mission at Limonade Creek in Saül, French Guiana, during October 10–25, 2013. Saül is an isolated village in the Amazonian rainforest (3°55′18′′N, 53°18′02′′W). 

Among the 7 patients, 6 were male; mean age was 32 ± 5 years. None of the patients were immunocompromised. The scientists stayed in Saül a mean of 17 (range 12–30) days. The mean time to symptom onset after they left Saül was 19 (range 0–50) days. The mean number of CL lesions was 2.3 (range 1–5). Lesions were localized mainly on lower limbs (11/14 lesions) but also appeared on upper limbs (2/14 lesions) and ears (1/14 lesions). CL was associated with nodular lymphangitis, adenitis, and superficial phlebitis of the affected limb in 2, 3, and 1 patient, respectively. No patients had mucosal involvement, fever, or decline in general health. 

Diagnosis of CL was clinically suggested and confirmed by microscope examination of skin scrapings, which revealed typical amastigotes, by a positive *Leishmania* species–specific PCR result, or both. *L. braziliensis* complex was diagnosed by using different molecular techniques, according to the laboratory, and then confirmation of *L. braziliensis* species was conducted by the French National Reference Center for Leishmaniasis on the basis of a putative translation initiation factor α-subunit gene sequence (*3*). *Leishmania* strain genotyping was performed to explore the epidemiology of the implicated strains. Four single-copy genomic loci were amplified from 5 of 7 patient samples; 1 of the samples had a parasite DNA content that was too low to genotype, and 1 was not analyzed. The genetic analysis of the 4 concatenated sequences showed 5 distinct and nonclustered genotypes (Figure). According to local protocols, patients were treated with 20 mg/kg of intramuscular meglumine antimoniate or with 18–38 mg/kg of intravenous liposomal amphotericin B; at publication time, the patients were still being followed.

**Figure:**
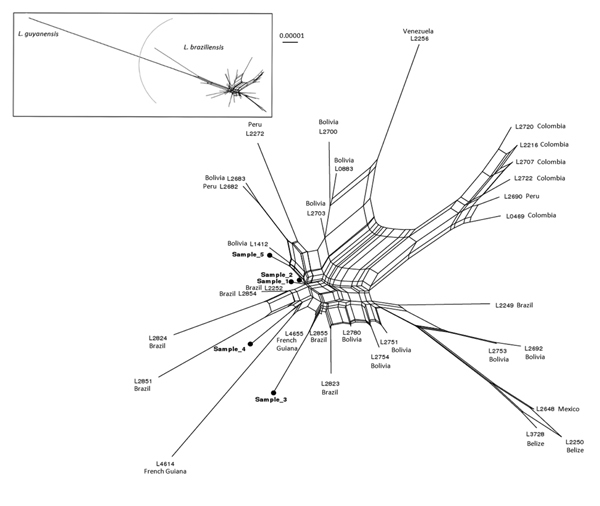
Data display network showing the genetic diversity of 32 *Leishmania braziliensis* (according to the multilocus enzyme electrophoresis–based taxonomy) compared with 5 strains from clinical samples (boldface) obtained from visitors to the Amazonian forest region of French Guiana. The strains were compared at 4 genomic loci (locus 03.0980, 10.0560, 31.0280 and 31.2610) as previously described (*3*). The concatenated nucleotide sequences (2,610 bp) were duplicated to avoid information loss due to heterozygous positions (e.g., A to AA or Y to CT). Neighbor-Net analysis was performed with SplitsTree version 4.11.3 (http://splitstree.org/) by using p-distances and equal edge lengths (*4*). Two *L. guyanensis* strains were used as an outgroup. The inset represents the genetic distance between L. guyanensis and L. braziliensis. Scale bar indicates evolutionary distance.

This outbreak of *L. braziliensis* CL in French Guiana raises the question of an overall increase in the incidence of this *Leishmania* species. Until now, outbreaks of *L. braziliensis* infection have been observed in Argentina, Brazil, Panama, and Venezuela but not Guiana (*5*–*7*). In French Guiana, changes in the epidemiology of CL have been observed since 2006; the emergence of *L. braziliensis*, *L. amazonensis*, and *L. lainsoni* infections represented 8.8%, 2.6%, and 1.4%, respectively, of the diagnosed CL cases (*8*). This trend could be due either to an increase of *L. braziliensis* prevalence in the forests of Guiana or to a greater presence of humans (e.g., military personnel, scientists, and tourists) in deep forest areas with hot spots of transmission. Favorable environmental conditions in a well-delimited zoonotic microfocus hot spot might have contributed to this high rate of transmission. However the relative genetic diversity of strains we observed among the 5 analyzed patients was unexpected, given the relatively small spatial and temporal scale of the transmission area, and indicates that the reservoirs in this restricted area were infested by distinct genotypes. Development of a peridomestic cycle, perhaps with specific reservoirs (pets) and vectors, cannot be excluded in the Saül area.

This case series suggests that caution should be taken in the diagnosis and treatment of CL in patients returning from the Amazonian rainforest, and a species-specific approach based on molecular identification should be proposed to provide appropriate medical management (*9*). Indeed, although *L. braziliensis* parasites cause <10% of CL acquired in French Guiana, this species is noteworthy for its involvement of the mucous membranes of the lips, nose, soft palate, or larynx. Also, *L. braziliensis* parasites usually fail to respond to pentamidine isethionate, the first-line treatment of *L. guyanensis* CL in French Guiana; instead, treatment of *L. braziliensis* infection relies on parenteral meglumine antimoniate or liposomal amphotericin B (*1*).

In summary, the geographic extension of and numeric increase in *L. braziliensis* cases in the Guiana ecoregion complex, as observed in the rest of South America, are worrisome, and continuous epidemiologic surveillance is needed. Infection with *L. braziliensis*, which is emerging and has potential to disseminate, must be considered in cases of CL acquired in this region. These issues have key implications for leishmaniasis treatment, which should be directed to the identified species (*10*).
